# Identification of genomic differences between *Campylobacter jejuni *subsp. *jejuni *and *C. jejuni *subsp. *doylei *at the *nap *locus leads to the development of a *C. jejuni *subspeciation multiplex PCR method

**DOI:** 10.1186/1471-2180-7-11

**Published:** 2007-02-28

**Authors:** William G Miller, Craig T Parker, Sekou Heath, Albert J Lastovica

**Affiliations:** 1 Produce Safety and Microbiology Research Unit, Agricultural Research Service, U.S. Department of Agriculture, Albany, CA 94710, USA; 2 Department of Biotechnology, University of the Western Cape, Bellville, South Africa

## Abstract

**Background:**

The human bacterial pathogen *Campylobacter jejuni *contains two subspecies: *C. jejuni *subsp. *jejuni *(*Cjj*) and *C. jejuni *subsp. *doylei *(*Cjd*). Although *Cjd *strains are isolated infrequently in many parts of the world, they are obtained primarily from human clinical samples and result in an unusual clinical symptomatology in that, in addition to gastroenteritis, they are associated often with bacteremia. In this study, we describe a novel multiplex PCR method, based on the nitrate reductase (*nap*) locus, that can be used to unambiguously subspeciate *C. jejuni *isolates.

**Results:**

Internal and flanking *napA *and *napB *primer sets were designed, based on existing *C. jejuni *and *Campylobacter coli *genome sequences to create two multiplex PCR primer sets, *nap *mpx1 and *nap *mpx2. Genomic DNA from 161 *C. jejuni *subsp. *jejuni *(*Cjj*) and 27 *C. jejuni *subsp. *doylei *(*Cjd*) strains were amplified with these multiplex primer sets. The *Cjd *strains could be distinguished clearly from the *Cjj *strains using either *nap *mpx1 or mpx2. In addition, combination of either *nap *multiplex method with an existing *lpxA *speciation multiplex method resulted in the unambiguous and simultaneous speciation and subspeciation of the thermophilic Campylobacters. The *Cjd nap *amplicons were also sequenced: all *Cjd *strains tested contained identical 2761 bp deletions in *napA *and several *Cjd *strains contained deletions in *napB*.

**Conclusion:**

The *nap *multiplex PCR primer sets are robust and give a 100% discrimination of *C. jejuni *subspecies. The ability to rapidly subspeciate *C. jejuni *as well as speciate thermophilic *Campylobacter *species, most of which are pathogenic in humans, in a single amplification will be of value to clinical laboratories in strain identification and the determination of the environmental source of campylobacterioses caused by *Cjd*. Finally, the sequences of the *Cjd napA *and *napB *loci suggest that *Cjd *strains arose from a common ancestor, providing clues as to the potential evolutionary origin of *Cjd*.

## Background

*Campylobacter *spp. are a common cause of acute bacterial gastroenteritis in humans [1,2]. The majority of campylobacterioses are caused by *C. jejuni *and are linked primarily to untreated water or consumption of poultry and raw milk [2]. *C. jejuni *has been divided into two subspecies: *C. jejuni *subsp. *jejuni *(*Cjj*) and *C. jejuni *subsp. *doylei *(*Cjd*). *Cjd *strains were isolated originally as "gastric campylobacter-like organisms type 2 (GCLO2)" from human gastric biopsies [4] and "nitrate-negative campylobacter-like organisms (NNC)" from Australian pediatric patients with gastroenteritis [5]. As the "NNC" designation suggests, the characteristic feature of *Cjd*, used commonly to distinguish *Cjd *strains from *Cjj *strains, is the inability to reduce nitrate. Other phenotypic characteristics, such as variable growth at 42°C [6], high susceptibility to cephalothin [6], and the absence of γ-glutamyl transferase (GGT) and L-arginine arylamidase activity [7] have been associated also with *Cjd*; however, like *Cjj*, all *Cjd *strains are hippuricase positive.

*Cjd *strains also differ from *Cjj *in two other clinically-related aspects: first, *Cjd *strains can be found throughout the gastrointestinal tract, leading to both gastritis [4,8] and enteritis [6,9-11], and are obtained often from pediatric patients [6,9,11,12]. Second, in South Africa, unlike *Cjj *strains, *Cjd *strains are isolated more often from blood cultures than from stool cultures [3]; *Cjd *was isolated from 24% of the *Campylobacter*-positive blood cultures [3], in contrast to 7.7% of the *Campylobacter*-positive stool cultures, obtained at Red Cross Children's Hospital, Cape Town, during the years 1990–2005. Additionally, Morey reported that *Cjd *was isolated from 85.2% of *Campylobacter*/*Helicobacter*-related bacteremia cases in Australia during a five-year period [12].

In some parts of the world, notably South Africa, *Cjd *strains represent a significant proportion of the total campylobacters isolated from human clinical samples; 16% of the non-*Cjj*/*coli Campylobacter *strains isolated in Cape Town, South Africa were *Cjd*. However, despite the unusual clinical symptomatology and relatively high prevalence in certain parts of the world, *Cjd *is generally isolated infrequently and few strains exist (compared to *Cjj*) for this subspecies. One possible reason is that many clinical laboratories do not characterize *Campylobacter *isolates past the genus level, much less subspeciate *C. jejuni *isolates as *Cjj *or *Cjd*. It is also likely that both the susceptibility of *Cjd *to cephalothin and variable growth at 42°C prevents the isolation of a substantial number of *Cjd *strains under normal *Cjj *isolation conditions; the *Cjd *strains isolated in South Africa were obtained using the Cape Town Protocol [3] which uses passive filtration through a 0.65 μM membrane filter, growth at 37°C and no antibiotic selection.

Another factor in the lack of subspeciation characterization of *C. jejuni *strains is the absence of a rapid test to distinguish *Cjj *from *Cjd*. Phenotypic characterization of *Cjd*, based on the absence of nitrate reductase activity, remains the primary means of identification; however, such nitrate reductase assays require a large number of cells and can take 24–48 h. Additionally, nitrate reductase assays require pure cultures; mixtures of *Cjj *and *Cjd *cells would type as nitrate^+^/*Cjj*.

Many commonly used molecular-based *Campylobacter *detection methods cannot be used to subspeciate *C. jejuni *due to the high similarity between the two subspecies. Molecular-based methods that can subspeciate *C. jejuni *do exist, based on hybridization [7], amplified fragment length polymorphism fingerprinting [13,14], or characterization of the 16S/23S rDNA internal spacer region [15]; however, these methods are lengthy or require specialized and costly equipment. In this study we present a simple, novel multiplex PCR method that can be used to unambiguously subspeciate *C. jejuni*.

## Results

### Development of the *napAB *multiplex subspeciation PCR

Preliminary results from *C. jejuni *DNA microarray experiments, using *Cjd *strain RM2095 as a tester strain, indicated that, in addition to other loci, the *napA *and *napB *genes in strain RM2095 were either absent or highly divergent with respect to *Cjj napA *and *napB *(Parker et al., submitted for publication). Since *napA *and *napB *encode the large and small subunits of nitrate reductase, respectively, this was not unexpected, given the absence of nitrate reductase activity in RM2095. Thus, the microarray results suggested that the *nap *phenotype in RM2095 was due most likely to deletions in *napA *and/or *napB *and that these results might be extended to *Cjd *in general; a loss of function in either subunit would result in a loss of enzyme activity. Therefore, an obvious target for a subspeciation multiplex PCR would be the *nap *locus. The *nap *locus in several *Campylobacter *species consists of six genes (in order): *napA*, *napG*, *napH*, *napB*, *napL*, and *napD*. Upstream of *napA *in *Cjj *and *C. coli *is the *tpx *gene, encoding a thiol peroxidase. The conservation of gene order and high nt identity between *C. jejuni *and *C. coli *at this locus suggested that primers designed to amplify both species should also amplify *Cjd *strains.

**Figure 1:**
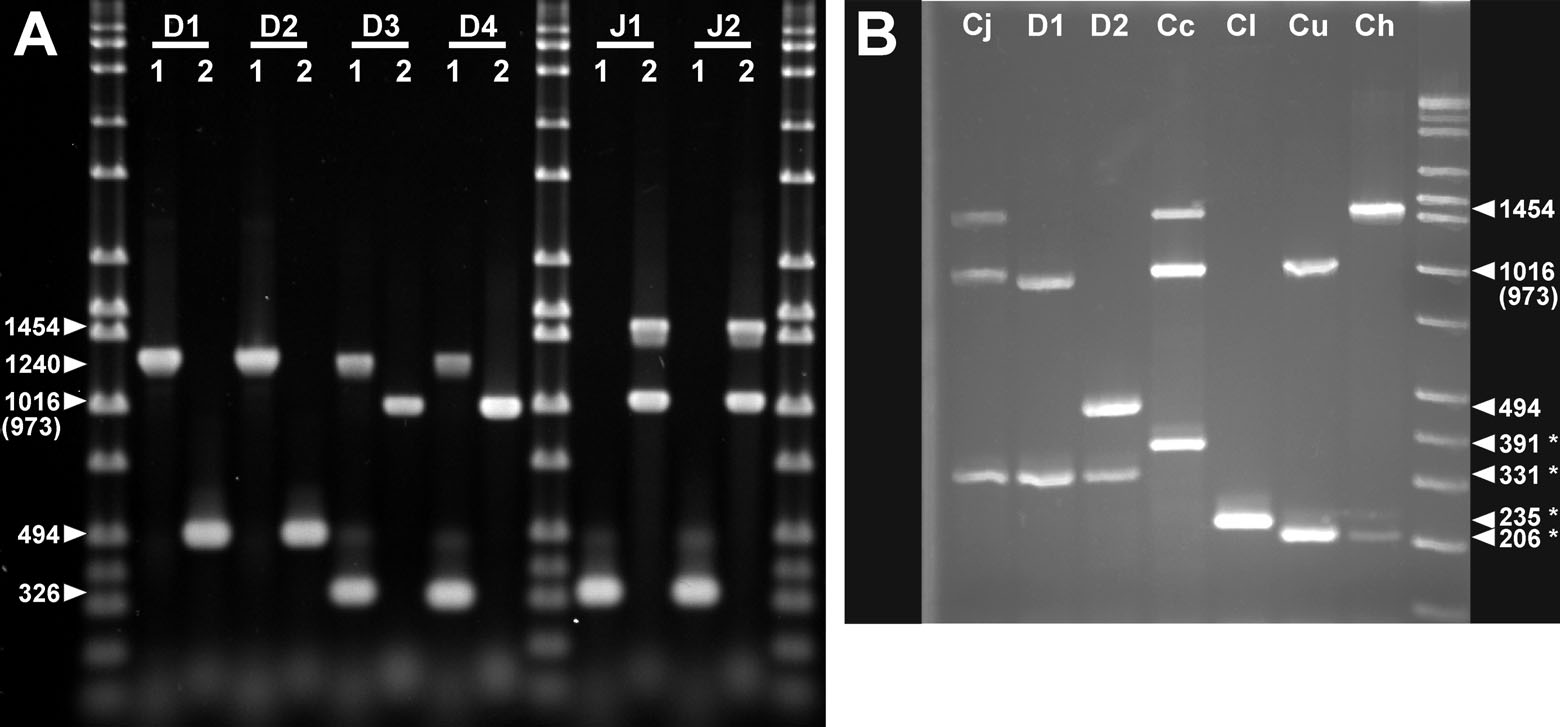
**Identification of *C. jejuni *subsp. *doylei *strains by multiplex PCR**. **A. ***napAB *multiplex PCR. *C. jejuni *genomic DNAs were amplified with either the *nap *mpx 1 ("1") or mpx 2 ("2") primer sets; the PCRs were run on a 1% agarose gel. D1: *Cjd *strain RM2095; D2: *Cjd *strain 269.97; D3: *Cjd *strain RM2096; D4: *Cjd *strain CCUG 18266; J1: *Cjj *strain NCTC 11168; J2: *Cjj *strain RM1221. Band sizes in bp are indicated on the left side of the gel. **B. **Combination speciation and subspeciation PCR. *Campylobacter *genomic DNAs were amplified with the combined *nap *mpx 2 and thermophilic *lpxA Campylobacter *speciation primer sets; PCRs were run on a 2% agarose gel. Cj: *Cjj *strain NCTC 11168; D1: *Cjd *strain CCUG 18266; D2: *Cjd *strain 269.97; Cc: *C. coli *strain RM2228; Cl: *C. lari *strain RM2100; Cu: *C. upsaliensis *strain RM3195; Ch: *C. helveticus *strain ATCC 51209. Band sizes in bp are indicated on the right side of the gel. Bands resulting from the speciation primer sets are indicated with an asterisk (*).

Therefore, the sequences of the *nap *regions (*tpx *to *napD*) of three strains (*Cjj *NCTC 11168, *Cjj *RM1221 and *C. coli *RM2228), obtained from the genome sequence for each strain [GenBank: AL111168, GenBank: CP000025, and GenBank: AAFL00000000, respectively], were aligned and four primer pairs were designed to regions of high conservation within the alignment: primers internal to and flanking *napA*, and primers internal to and flanking *napB*. The primers were organized into two multiplex primer sets: *nap *mpx1 and *nap *mpx2 (Table 1). A representative gel illustrating the *nap *mpx1 and *nap *mpx2 amplicons is presented in Fig. 1A.

Since *napA *is quite large (~2.8 kb), *Cjj *strains would not be expected to amplify with the *napA *flanking primers under standard PCR conditions; however, both *Cjj *strains did amplify with the *napA *internal primer set (1454 bp: Fig. 1A). The *Cjd *strains did not amplify with the *napA *internal primer set but did amplify with the *napA *flanking primers. The resulting amplicon (1240 bp: Fig 1A) was reduced in size from that predicted for *Cjj *by about 2.8 kb, indicating the presence of a deletion within *napA*; amplification with different sets of flanking primers indicated that this deletion extended into *napG *(data not shown). Noteworthy also was the fact that the *Cjd *strains could be divided into two classes, differentiated by an apparent presence or absence of *napB*. The "*napA^-^napB^+^*" strains (D3, D4: Fig 1A), termed *Cjd1*, amplified with the *napB *internal primer set (326 bp: Fig. 1A) and gave a full length amplicon (973 bp: Fig 1A) with the *napB *flanking primer set. The "*napA^-^napB^-^*" strains (D1, D2: Fig 1A), termed *Cjd2*, gave a reduced length amplicon (494 bp: Fig 1A) with the *napB *flanking primer set, indicating the presence of a *napB *deletion. Both classes would be unable to reduce nitrate, based on the common *napA *deletion.

### Validation of the *nap *multiplex PCR using additional *Campylobacter *genomic DNAs

To test the specificity of the *nap *mpx primer sets, an additional 20 human clinical *Cjd *strains and 158 human clinical, animal and environmental *Cjj *strains were amplified using the *nap *mpx2 primer set. The PCR results were identical to those presented in Fig. 1A [Table S1, see additional file 1] with two exceptions. The HS:63 Penner serotype reference strain, listed originally as *Cjj*, amplified as a *Cjd2 *isolate; however, this serotype has been seen previously in *Cjd *isolates [16] and therefore this strain may have been misidentified as a *Cjj*. Also, one *Cjd *strain amplified as a *Cjj *isolate; a second nitrate reductase test on this strain indicated that the strain was mis-typed originally.

Genomic DNA from the *C. coli *strain RM2228 amplified also with the *nap *mpx2 primer set; the resulting banding pattern was identical to that of *Cjj *(data not shown). Consequently, representatives of the remaining non-*jejuni*/*coli Campylobacter *spp. were amplified using the *nap *mpx2 primer set. Genomic DNA from five of these species [*C. upsaliensis *(RM3195), *C. lari *(RM2100), *C. helveticus *(RM3228),* C. sputorum *(RM3237) and *C. mucosalis *(RM3234)] amplified, although the banding patterns were distinct from those in Fig. 1A [Table S1, see additional file 1]. Therefore, 107 additional strains of these five species and 10 additional *C. coli *strains were amplified using both primer sets. All of the *C. coli *strains and some of the *C. upsaliensis *strains produced both of the *Cjj*-characteristic 1454 bp and 1016 bp bands following amplification with *nap *mpx2 (11/11 and 23/72, respectively; [Table S1, see additional file 1]). Moreover, the banding patterns for many of these 34 *C. coli *and *C. upsaliensis *strains were similar to those seen in Fig. 1A for both multiplex primer sets; therefore, *C. coli *and *C. upsaliensis *strains could be confused potentially with *Cjj *isolates.

### Combination of the *nap *and *lpxA *multiplex PCRs gives unambiguous speciation and subspeciation of thermophilic campylobacters

If the *nap *multiplex PCRs were performed on strains already speciated as *C. jejuni*, then the non-specificity of the *nap *PCR towards *C. coli *and *C. upsaliensis *would not be of concern. However, in many instances, food, animal and environmental samples contain multiple *Campylobacter *species, especially *C. jejuni *and *C. coli*. In this case, the non-specificity of the *nap *PCR would not permit accurate identification. A PCR speciation method for thermophilic *Campylobacter *spp, based on the lipid A gene *lpxA*, was published recently [17]. Therefore, to extend the discriminatory power of the *nap *subspeciation PCR method, we tested whether unambiguous speciation and subspeciation of thermophilic *Campylobacter *spp. would be feasible by combining the *lpxA *and *nap *mpx PCR methods. Genomic DNAs from five thermophilic *Campylobacter *species were amplified with the nine-primer *nap*/*lpxA *primer set. Unique banding patterns were obtained for all species/subspecies (Fig. 1B), indicating that unambiguous speciation and subspeciation of thermophilic campylobacters can be accomplished with only one amplification.

### Characterization of the *Cjd napB *and *napA *loci

As described above, the *Cjd *strains were subdivided into two groups based on the apparent presence (*Cjd1*) or absence (*Cjd2*) of *napB*. To characterize the *Cjd napB *genes, the amplicons obtained with the *napB *flanking primers were sequenced. The *napB *loci of *Cjd2 *strains RM2095, RM3782 and SSI 5384 contained an approx. 520 bp deletion extending from nt -23 to nt 495 (data not shown). All three deletions had identical endpoints; however, single nucleotide polymorphisms present in the *napB *amplicon sequences indicated that all three strains were genetically distinct. Interestingly, three of the four *Cjd1 *strains contained an extra G at *napB *nt 15, resulting in a truncated NapB; thus, of the eight *Cjd1 *and *Cjd2 *strains, only RM2096 would be predicted to encode a full-length, and presumably functional, NapB protein.

Similarly, the amplicons obtained with the *napA *flanking primers were sequenced. As with *napB*, single nucleotide polymorphisms are present also in the amplicon sequences of five of the eight strains, indicating that these five strains are different. The remaining strains, ATCC 49350, ATCC 49351 and CCUG 18266, have identical *napA *sequences. As predicted, all eight *Cjd napA *genes contain 2761 bp deletions that extend from *napA *(nt 493) into *napG*; unexpectedly, however, all eight deletions have identical endpoints. Amplification and sequencing of the *napA *loci from an additional 19 *Cjd *isolates also revealed the presence of identical *napA *deletions.

## Discussion

The apparent absence of the genes encoding nitrate reductase (*napA *and *napB*) in *Cjd *strain RM2095 correlated well with the nitrate reduction-negative phenotype characteristic of this subspecies. Thus, we reasoned that a potential subspeciation marker for *C. jejuni *would be the *nap *locus, specifically *napA *and *napB*, and we developed therefore a *napA*/*B *multiplex PCR method to subspeciate *C. jejuni*. Amplification results indicate that this *nap *multiplex PCR method, using internal and flanking primer sets for *napA *and *napB*, can be used to subspeciate unambiguously *C. jejuni*: *Cjd *strains (D1-4: Fig. 1A) can be distinguished readily from *Cjj *strains (J1-2: Fig. 1A). Moreover, different banding patterns were observed with *Cjj *and *Cjd *using *nap *mpx1 or *nap *mpx2, suggesting that either multiplex primer set is sufficient to subspeciate *C. jejuni*. The almost total concordance between the subspecies identification of 188 *C. jejuni *strains and the multiplex PCR results indicates that the new multiplex PCR is robust and can be used successfully to subspeciate *C. jejuni*. One strain, the Penner HS:63 reference strain, classified originally as *Cjj*, was identified as *Cjd *by our assay, suggesting a potential flaw in the PCR method. However, identification of a strain as *Cjd *by our method requires a deletion in *napA *and/or *napB*. Since a deletion in either of these two genes would lead necessarily to a loss of nitrate reductase activity, *C. jejuni *strains containing such defects would be, by definition *Cjd*, regardless of the original classification. Therefore, the Penner HS:63 reference strain was either not subspeciated or was subspeciated incorrectly. Finally, combination of the *napA*/*B *multiplex method with an *lpxA *multiplex method designed to speciate thermophilic Campylobacters permits the speciation and subspeciation of thermophilic Campylobacters with a single amplification reaction. Combination of the two methods is especially important when the species identification of a *Campylobacter *strain is unknown, as *C. coli *and *C. upsaliensis *strains may be confused potentially with *Cjj *(although not *Cjd*) strains if the *nap *multiplex method is used solely.

Currently, *Cjd *strains are subspeciated using the nitrate reductase assay, an assay used initially also in this study to identify *Cjd *strains. Although the nitrate reductase assay is relatively easy to perform, the multiplex PCR methods described here have several advantages over the nitrate reductase assay. First, subspeciation of *C. jejuni *strains, as well as the speciation/subspeciation of thermophilic Campylobacters, can be accomplished with this assay in a matter of hours, compared to the 2–3 days necessary for the nitrate reductase assay, and in a single amplification reaction. Second, the nitrate reductase assay requires a pure culture. Mixtures of *Campylobacter *strains of the same species or strains representing different species occur often during isolation [3,18,19] and would be problematic with regards to phenotypic assays; mixtures of *Cjj *and *Cjd *cells would type as *Cjj*. In contrast, the multiplex PCR method does not require a pure culture and would identify the species/subspecies comprising such a mixture. In fact, the multiplex speciation method identified successfully each species in a mixture of four thermophilic *Campylobacter *genomic DNAs (data not shown). Third, the nitrate reductase assay requires often more than a single colony for accurate identification. However, the multiplex PCR method requires at most only a single colony. Indeed, 19 of the *Cjd *genomic DNAs used in this study were obtained by boiling a single storage bead in 10 mM Tris pH 8.0, indicating that strains in storage at -80°C do not even have to be grown out to be characterized. Finally, the nitrate reductase assay does lead occasionally to false identification. One of the strains in this study, identified initially as *Cjd*, was determined by the multiplex PCR method to be *Cjj*. A repeat of the nitrate reductase assay on this strain indicated that it was, in fact, *Cjj *and that the original nitrate reductase identification was in error. Taken together, these results indicate that the *napA*/*B *multiplex PCR method described here is a valuable tool for clinical and research laboratories and can be used successfully to subspeciate rapidly *C. jejuni *strains and speciate thermophilic Campylobacters when combined with the *lpxA *multiplex PCR method.

An unexpected outcome of this study arose from the sequencing of the *Cjd napA *and *napB *amplicons. Some *Cjd *strains contained deletions in *napB *(Cjd2) and some contained apparently full-length *napB *genes (*Cjd1*), although point mutations in many of the *Cjd1 napB *genes would result in non-functional proteins; however, all 27 characterized *Cjd *strains contained identical *napA *deletions of 2761 bp. Based on the presence of these identical *napA *deletions, it appears that the *Cjd *strains, isolated on four continents over at least two decades, share a common ancestor. Furthermore, as the defining phenotype of *Cjd *is the absence of nitrate reduction, the evidence suggests strongly that *Cjd *arose from a single evolutionary event, i.e. the *napA *deletion, with the divergence at *napB *occurring somewhat later; deletion of the gene encoding the large subunit of nitrate reductase would remove the constraint maintaining a functional small subunit-encoding gene. Obviously, further experiments will be necessary to validate this hypothesis. Such experiments would entail the isolation and characterization of additional *Cjd *isolates, facilitated by the multiplex PCRs described above. Although the results presented here will need to be investigated further, they nevertheless provide intriguing insights into the origin of this subspecies.

## Conclusion

*Campylobacter jejuni *subsp. *doylei *strains are isolated infrequently. They are isolated primarily from human clinical patients and often from blood cultures, suggesting that they may be more pathogenic than *Cjj *strains. However, little is known about the *doylei *subspecies, especially with regards to the environmental reservoirs for this organism; no animal host has been yet identified for *Cjd *[20]. *Cjj *strains are isolated often from avian hosts. The absence of *Cjd *from avian sources, such as poultry, may be due to the lower maximum growth temperature (37°C) for this subspecies. This lower growth temperature may restrict *Cjd *strains to other hosts with lower normal body temperatures, such as swine and domestic pets. It may influence also the isolation of *Cjd *at the clinical level since enrichment and selection of *C. jejuni *as a whole is normally performed at 42°C, precluding the isolation of most *Cjd *strains. Also, antibiotics used in typical *C. jejuni *isolation media may restrict the growth of some *Cjd *strains.

The multiplex PCR method presented in this study will enhance greatly the identification of *Cjd *strains from human clinical, veterinary and environmental samples. Since this PCR does not require large numbers of cells or pure cultures, *Cjd *strains can be identified even when they comprise only a small subset of the overall population. Removing the need for enrichment or selection would reduce the isolation bias that might minimize or eliminate detection of *Cjd *strains. Finally, we have demonstrated here that PCR identification of *Cjd *strains can be performed using only the cells coating bacterial storage beads from -80°C freezer stocks. Thus, strains identified only as *C. jejuni *can be re-assessed rapidly without the necessity of growing the strains out to purity. This was instrumental in identifying, for example, an HS:63 Penner serotype reference strain, identified initially as *Cjj*, as a *Cjd *strain; several other strains identified only as *C. jejuni *may be, in fact, *C. jejuni *subsp. *doylei*.

## Methods

### Bacterial strains, growth conditions and chemicals

*Campylobacter *strains used in this study are listed in Table 2. All *Cjd *strains were cultured routinely at 37°C under microaerophilic conditions (5% O_2_, 10% CO, and 85% N_2_) on Anaerobe basal agar (ABA; Oxoid, Basingstoke, UK) amended with 5% (v/v) laked horse blood (Hema Resource & Supply, Aurora, OR). All chemicals were purchased from Sigma-Aldrich (St. Louis, MO) or Fisher Scientific (Houston, TX). PCR enzymes and reagents were purchased from New England Biolabs (Beverly, MA) or Epicentre (Madison, WI). DNA sequencing chemicals and capillaries were purchased from Applied Biosystems (Foster City, CA). Sequencing and PCR oligonucleotides were purchased from MWG-Biotech (High Point, NC).

### Phenotypic characterization of *C. jejuni* subsp. *doylei* strains

To determine nitrate reduction in *C. jejuni*, nitrate disks and anaerobic nitrate reagents A and B (Remel, Lenexa, KS) were used. Zinc dust was obtained from BioMérieux. *Cjj *strain NCTC 11168 and *Cjd *strain ATCC 49350 were used as controls for the procedure. Each *Cjd *strain was streaked onto ABA agar supplemented with 5% horse blood and grown for 48 h as described above. A nitrate disk was then placed on a thick zone of growth on the plate and incubated for an additional 24 h under the same conditions. The nitrate disk was removed subsequently and placed in a sterile tube. One drop of both reagents A and B were added to the disk. Reduction of nitrate is indicated by a color change (clear to red); if no color change was observed after 3 min, zinc dust was added. A color change prior to the addition of zinc is indicative of *Cjj *and a color change only after addition of zinc is indicative of *Cjd*.

### Multiplex PCR

Genomic DNA from the *Cjd *strains in Table 2 was prepared using the Wizard Genomic DNA kit (Promega, Madison, WI) according to the manufacturer's protocols. Additional *Cjd *genomic DNAs were prepared by boiling single Microbank bacterial storage beads (Pro-Lab, Austin, TX) from freezer stocks for 5 min in 100 μl of 10 mM Tris pH 8.0. Genomic DNA from other *Campylobacter *strains was prepared as described [21]. *nap *multiplex PCRs were performed on a Tetrad thermocycler (Bio-Rad, Hercules, CA) with the following settings: 30 s at 94°C; 30 s at 53°C; 2 min at 72°C (30 cycles). Each amplification mixture contained 50 ng genomic DNA, 1× PCR buffer (Epicentre), 1× MasterAmp enhancer (Epicentre), 2.5 mM MgCl_2_, 250 μM each dNTP, 50 pmol each primer (Table 1), and 1 U *Taq *polymerase (New England Biolabs).* nap*/*lpxA *multiplex PCRs were performed under similar parameters, with a final MgCl_2_ concentration of 2.0 mM; additionally, 30 pmol of the KK2 primer (Table 1) and 10 pmol of the lpxA*C. coli*, lpxA*C. jejuni*, Cl0122 and lpxA*C. upsaliensis *primers (Table 1) were added to each reaction.

### DNA sequencing

Cycle sequencing reactions were performed on a Tetrad thermocycler using the ABI PRISM BigDye terminator cycle sequencing kit (version 3.1) and standard protocols. All extension products were purified on DyeEx 96 well plates (Qiagen). DNA sequencing was performed on an ABI PRISM 3130XL Genetic Analyzer (Applied Biosystems) using the POP-7 polymer and ABI PRISM Genetic Analyzer Data Collection and ABI PRISM Genetic Analyzer Sequencing Analysis software.

### GenBank accession numbers

The partial *Cjd napA *and *napB *sequences were submitted to GenBank and have the following accession numbers: ATCC 49350 *napA*: [GenBank:EF218724 ]; ATCC 49350 *napB*: [GenBank: EF218725]; ATCC 49351 *napA*: [GenBank: EF218726]; ATCC 49351 *napB*: [GenBank: EF218727]; RM2095 *napA*: [GenBank: EF218728]; RM2095 *napB*: [GenBank: EF218729]; RM2096 *napA*: [GenBank: EF218730]; RM2096 *napB*: [GenBank: EF218731]; RM3782 *napA*: [GenBank: EF218732]: RM3782 *napB*: [GenBank: EF218733]; SSI 5384 *napA*: [GenBank: EF218734]; SSI 5384 *napB*: [GenBank: EF218735]; CCUG 18266 *napA*: [GenBank: EF218736]; CCUG 18266 *napB*: [GenBank: EF218737]; 269.97 *napA*: [GenBank: EF218738]; 269.97 *napB*: [GenBank: EF218739].

## Authors' contributions

WGM and AJL designed the research project. WGM designed the *nap *mpx1 and mpx2 multiplex primer sets and was the principal author of the manuscript. CTP constructed the *C. jejuni *DNA microarray and performed the microarray experiments and analysis. SH performed all of the multiplex amplifications and sequenced all of the *napA *and *napB *amplicons. AJL collected the 21 South African *Cjd *strains and performed the initial nitrate reductase assays on these isolates. All authors approved and read the final manuscript.

## Supplementary Material

Additional File 1 Table S1. *Campylobacter *strains used to validate the *nap *multiplex PCR assay. The 321 *Campylobacter *strains used in the *nap *multiplex PCR validation are briefly described, including source/location of isolation and serotype, where available. Additionally, for each strain, the amplicon sizes (in bp, when present) for the *nap *and *lpxA *multiplex PCRs are provided. Click here for file

## Figures and Tables

**Table 1:** *nap *and *lpxA *multiplex primers

***nap *mpx1:**		
napAL2	Flanking	5' CTT TAG AAG GGC TTT TAG CTC GTG C 3'
napAR4	Flanking	5' ATT TCC CTG CAA GAT AAA ATC TGT AGC 3'
napBIF	Internal	5' AGA AAA GCA AGT TTA GAA AAT GAA AAT AA 3'
napBIR	Internal	5' GCA TCA CTT TGT GGA ACA TGA CA 3'
***nap *mpx2:**		

napAIF3	Internal	5' TAG AAC AAA TAA TAT CGA TCC AAA TGC 3'
napAIR3	Internal	5' AAA AGT GTA TCA TCT TCG CTA TAA CCC 3'
napBL	Flanking	5' GGA ATG ATA CAT AGA GGG ATT ATT TTT G 3'
napBR	Flanking	5' AAT TTC ACC TTT ATC AGT GCC TAT ATA 3'
***lpxA***:		

KK2		5' CAA TCA TGW GCN ATA TGR CAA TAN GCC 3'
lpxA*C. coli*		5' AGA CAA ATA AGA GAG AAT CAG 3'
lpxA*C. jejuni*		5' ACA ACT TGG TGA CGA TGT TGT A 3'
Cl0122		5' CTT ACC AAA TGT TAA AAT AGG C 3'
lpxA*C. upsaliensis*		5' AAG TCG TAT ATT TTC YTA CGC TTG TGT G 3'

lpxA*C. coli*, lpxA*C. jejuni *and lpxA*C. upsaliensis *are from Klena et al. [17]. KK2 and Cl0122 are derived from lpxARKK2m and lpxA*C. lari*, respectively [17]; the Cl0122/KK2 amplicon is 2 bp longer than the respective lpxARKK2m/lpxA*C. lari *amplicon.

**Table 2:** *Campylobacter *strains used in this study

**Strain**^**a**^	**Description**	**Location**	**Reference or source**
NCTC 11168	*Campylobacter jejuni *subsp. *jejuni*; Lior 4, Penner HS:2	UK	Human clinical isolate; [22].
RM1221	*Campylobacter jejuni *subsp. *jejuni*; Penner HS:53	USA	Chicken carcass; [19].
81–176	*Campylobacter jejuni *subsp. *jejuni*; Penner HS:23,36	USA	Human clinical isolate; [23, 24].
ATCC 49350	*Campylobacter jejuni *subsp. *doylei*	Germany	Human clinical isolate; [4].
ATCC 49351	*Campylobacter jejuni *subsp. *doylei*	Australia	Human clinical isolate; [25].
CCUG 18266	*Campylobacter jejuni *subsp. *doylei*	Germany	Human clinical isolate; [4].
RM2095	*Campylobacter jejuni *subsp. *doylei*	USA	Mabel Nicholson; human blood.
RM2096	*Campylobacter jejuni *subsp. *doylei*	USA	Mabel Nicholson; human clinical isolate.
RM3782	*Campylobacter jejuni *subsp. *doylei*	South Africa	Human clinical isolate.
SSI 5384	*Campylobacter jejuni *subsp. *doylei*	Denmark	Stephen On; human clinical isolate.
269.97	*Campylobacter jejuni *subsp. *doylei*	South Africa	Human blood.
RM2228	*Campylobacter coli*	USA	Chicken carcass; [26].
RM2100	*Campylobacter lari*	USA	Human clinical isolate; [26].
RM3195	*Campylobacter upsaliensis*	South Africa	Human clinical isolate; [26].
ATCC 51209^T^	*Campylobacter helveticus*	Switzerland	Feline isolate.

**a. **^T^: type strain.
