# Supplementary material for: Identification of genomic differences between Campylobacter jejuni subsp. jejuni and C. jejuni subsp. doylei at the nap locus leads to the development of a C. jejuni subspeciation multiplex PCR method
Source: BMC Microbiol. 2007 Feb 28;7:11. doi: 10.1186/1471-2180-7-11 (PMC1820782; doi:10.1186/1471-2180-7-11)
Supplement: Additional File 1 — Table S1. Campylobacter strains used to validate the nap multiplex PCR assay. The 321 Campylobacter strains used in the nap multiplex PCR validation are briefly described, including source/location of isolation and serotype, where available. Additionally, for each strain, the amplicon sizes (in bp, when present) for the nap and lpxA multiplex PCRs are provided. [file 1471-2180-7-11-S1.pdf]

Table S1: *Campylobacter* strains used to validate the *nap* multiplex PCR assay.

RM#	Other name	Organism	Source	Location	Description	*nap* mpx2 bands[a]	*lpxA* band[a]
1051	ATCC 43479	*C. coli*	Human	Canada	HS:30	1454, 1016	391
1166	Cc T21	*C. coli*	Chicken	Unknown	Lior 21	1454, 1016	391
1505	ATCC 49299	*C. coli*	Unknown	Unknown	HS:61	1454, 1016	391
1530	ATCC 43476	*C. coli*	Sheep	USA	HS:25	1454, 1016	391
1531	ATCC 43478	*C. coli*	Marmoset	Unknown	HS:28	1454, 1016	391
1532	ATCC 43482	*C. coli*	Human	USA	HS:46	1454, 1016	391
1533	ATCC 43485	*C. coli*	Human	USA	HS:49	1454, 1016	391
1857	D2611	*C. coli*	Human	Unknown		1454, 1016	391
1858	D2699	*C. coli*	Human	Unknown		1454, 1016	391
1865	D118	*C. coli*	Human	Unknown		1454, 1016	391
2228		*C. coli*	Chicken	USA	HS:34; Sequence strain	1454, 1016	391
3273	ATCC 33237	*C. concisus*	Human	Unknown	Type strain	None	None
3270	ATCC 35224	*C. curvus*	Human	USA	Type strain	None	None
2088	D233	*C. fetus* subsp. *fetus*	Human	USA		None	None
3226	ATCC 19438	*C. fetus* subsp. *venerealis*	Bovine	Unknown	Type strain	None	None
3268	ATCC 33236	*C. gracilis*	Human	Unknown	Type strain	None	None
3228	ATCC 51209	*C. helveticus*	Feline	Switzerland	Type strain	1454	206
3229	ATCC 51210	*C. helveticus*	Feline	Switzerland		1454	206
3807		*C. helveticus*	Feline	USA (CA)		1454	206
4087	CCUG 30566	*C. helveticus*	Feline	Switzerland		1454	206
4088	CCUG 34016	*C. helveticus*	Feline	Sweden		1454	206
4139	CCUG 34042	*C. helveticus*	Feline	Sweden		1454	206
4140	CCUG 30563	*C. helveticus*	Feline	Switzerland		1454	206
4141	CCUG 30564	*C. helveticus*	Feline	Switzerland		1454	206
4142	CCUG 30683	*C. helveticus*	Feline	Switzerland		1454	206
4093	176.96	*C. hyointestinalis* subsp. *hyointestinalis*	Human	S. Africa		None	None
4096	CCUG 27631	*C. hyointestinalis* subsp. *lawsonii*	Porcine	Sweden		None	None
1512	ATCC 49350	*C. jejuni* subsp. *doylei*	Human	Germany		973	331
1513	ATCC 49351	*C. jejuni* subsp. *doylei*	Human	Australia		973	331

2095	D2722	*C. jejuni* subsp. *doylei*	Human blood	USA		494	331
2096	D2781	*C. jejuni* subsp. *doylei*	Human	USA		973	331
3782	34.02	*C. jejuni* subsp. *doylei*	Human	S. Africa		494	331
4097	SSI 5384	*C. jejuni* subsp. *doylei*	Human	Denmark		494	331
4098	CCUG 18266	*C. jejuni* subsp. *doylei*	Human	Germany		973	331
4099	269.97	*C. jejuni* subsp. *doylei*	Human blood	S. Africa	HS:17; Sequence strain	494	331
	168.01	*C. jejuni* subsp. *doylei*	Human	S. Africa		494	331
	88.05	*C. jejuni* subsp. *doylei*	Human	S. Africa		494	331
	170.98	*C. jejuni* subsp. *doylei*	Human blood	S. Africa		494	331
	3.03	*C. jejuni* subsp. *doylei*	Human	S. Africa		494	331
	353.97	*C. jejuni* subsp. *doylei*	Human blood	S. Africa	HS:63	494	331
	Mf 62850	*C. jejuni* subsp. *doylei*	Human	S. Africa		494	331
	178.03	*C. jejuni* subsp. *doylei*	Human	S. Africa		494	331
	57.02	*C. jejuni* subsp. *doylei*	Human	S. Africa		494	331
	88.02	*C. jejuni* subsp. *doylei*	Human	S. Africa		494	331
	206.02	*C. jejuni* subsp. *doylei*	Human	S. Africa		494	331
	8.02	*C. jejuni* subsp. *doylei*	Human	S. Africa		494	331
	164.02	*C. jejuni* subsp. *doylei*	Human blood	S. Africa		494	331
	175.02	*C. jejuni* subsp. *doylei*	Human	S. Africa		494	331
	77.01	*C. jejuni* subsp. *doylei*	Human	S. Africa		1454, 1016	331
	341.94	*C. jejuni* subsp. *doylei*	Human	S. Africa		494	331
	327.93	*C. jejuni* subsp. *doylei*	Human	S. Africa		494	331
	362.97	*C. jejuni* subsp. *doylei*	Human blood	S. Africa		494	331
	340.93	*C. jejuni* subsp. *doylei*	Human	S. Africa		494	331
	317.95	*C. jejuni* subsp. *doylei*	Human	S. Africa		494	331
	260.95	*C. jejuni* subsp. *doylei*	Human	S. Africa		494	331
1045	ATCC 43429	*C. jejuni* subsp. *jejuni*	Human	Unknown	HS:1	1454, 1016	331
1046	ATCC 43430	*C. jejuni* subsp. *jejuni*	Calf	Unknown	HS:2	1454, 1016	331
1047	ATCC 43431	*C. jejuni* subsp. *jejuni*	Human	Unknown	HS:3	1454, 1016	331
1048	ATCC 43432	*C. jejuni* subsp. *jejuni*	Human	Canada	HS:4	1454, 1016	331
1049		*C. jejuni* subsp. *jejuni*	Human	Unknown		1454, 1016	331
1050	ATCC 43449	*C. jejuni* subsp. *jejuni*	Human	Canada	HS:23	1454, 1016	331
1052	ATCC 43456	*C. jejuni* subsp. *jejuni*	Human	Unknown	HS:36	1454, 1016	331
1155	CjT1	*C. jejuni* subsp. *jejuni*	Human	Canada	Lior 1	1454, 1016	331
1156	CjT2	*C. jejuni* subsp. *jejuni*	Human	Canada	Lior 2	1454, 1016	331

1158	CjT5	*C. jejuni* subsp. *jejuni*	Human	Canada	Lior 5	1454, 1016	331
1160	CjT7	*C. jejuni* subsp. *jejuni*	Human	Canada	Lior 7	1454, 1016	331
1163	CjT11	*C. jejuni* subsp. *jejuni*	Human	Israel	Lior 11	1454, 1016	331
1167	CjT28	*C. jejuni* subsp. *jejuni*	Human	Canada	Lior 28	1454, 1016	331
1170	NLEP 02-1971	*C. jejuni* subsp. *jejuni*	Chicken	USA		1454, 1016	331
1188		*C. jejuni* subsp. *jejuni*	Chicken	USA		1454, 1016	331
1221		*C. jejuni* subsp. *jejuni*	Chicken	USA (CA)	HS:53; Sequence strain	1454, 1016	331
1244	90A2737	*C. jejuni* subsp. *jejuni*	Human	USA		1454, 1016	331
1245	96A5046	*C. jejuni* subsp. *jejuni*	Human	USA		1454, 1016	331
1246	92A3120	*C. jejuni* subsp. *jejuni*	Human	USA		1454, 1016	331
1247	96A11074	*C. jejuni* subsp. *jejuni*	Human	USA		1454, 1016	331
1248	96A14504	*C. jejuni* subsp. *jejuni*	Human	USA		1454, 1016	331
1268	ATCC 33560	*C. jejuni* subsp. *jejuni*	Bovine	Unknown		1454, 1016	331
1285		*C. jejuni* subsp. *jejuni*	Chicken	USA		1454, 1016	331
1409		*C. jejuni* subsp. *jejuni*	Chicken	USA		1454, 1016	331
1413		*C. jejuni* subsp. *jejuni*	Chicken	USA		1454, 1016	331
1437		*C. jejuni* subsp. *jejuni*	Chicken	USA		1454, 1016	331
1443		*C. jejuni* subsp. *jejuni*	Chicken	USA		1454, 1016	331
1449		*C. jejuni* subsp. *jejuni*	Chicken	USA		1454, 1016	331
1464		*C. jejuni* subsp. *jejuni*	Chicken	USA		1454, 1016	331
1477	D445	*C. jejuni* subsp. *jejuni*	Human	USA		1454, 1016	331
1478	D226	*C. jejuni* subsp. *jejuni*	Human	USA		1454, 1016	331
1479	EDL18	*C. jejuni* subsp. *jejuni*	Human	USA		1454, 1016	331
1480	D1117	*C. jejuni* subsp. *jejuni*	Human	USA		1454, 1016	331
1501		*C. jejuni* subsp. *jejuni*	Chicken	USA		1454, 1016	331
1503	ATCC 43462	*C. jejuni* subsp. *jejuni*	Human	Canada	HS:43	1454, 1016	331
1507	LCDC 17384	*C. jejuni* subsp. *jejuni*	Human	Germany	Lior 16, HS:10	1454, 1016	331
1508	LCDC 17385	*C. jejuni* subsp. *jejuni*	Human	Germany	Lior 11, HS:53	1454, 1016	331
1510	LCDC 17402	*C. jejuni* subsp. *jejuni*	Human	Japan	HS:19, GBS isolate	1454, 1016	331
1511	LCDC 17403	*C. jejuni* subsp. *jejuni*	Human	Japan	HS:19, GBS isolate	1454, 1016	331
1516	ATCC 33560	*C. jejuni* subsp. *jejuni*	Human	USA		1454, 1016	331
1551	ATCC 43433	*C. jejuni* subsp. *jejuni*	Human	Unknown	HS:5	1454, 1016	331
1552	ATCC 43434	*C. jejuni* subsp. *jejuni*	Human	Canada	HS:6	1454, 1016	331
1553	ATCC 43435	*C. jejuni* subsp. *jejuni*	Human	Unknown	HS:7	1454, 1016	331
1554	ATCC 43436	*C. jejuni* subsp. *jejuni*	Human	Canada	HS:8	1454, 1016	331

1555	ATCC 43437	*C. jejuni* subsp. *jejuni*	Goat	Unknown	HS:9	1454, 1016	331
1556	ATCC 43438	*C. jejuni* subsp. *jejuni*	Human	Unknown	HS:10	1454, 1016	331
1844	D135	*C. jejuni* subsp. *jejuni*	Human	Canada		1454, 1016	331
1845	D140	*C. jejuni* subsp. *jejuni*	Human	Japan		1454, 1016	331
1846	D141	*C. jejuni* subsp. *jejuni*	Goat	Canada		1454, 1016	331
1847	D142	*C. jejuni* subsp. *jejuni*	Sheep	USA		1454, 1016	331
1849	D781	*C. jejuni* subsp. *jejuni*	Chicken	USA		1454, 1016	331
1850	D983	*C. jejuni* subsp. *jejuni*	Chicken	USA		1454, 1016	331
1851	D1038	*C. jejuni* subsp. *jejuni*	Chicken	USA		1454, 1016	331
1852	D1420	*C. jejuni* subsp. *jejuni*	Chicken	USA		1454, 1016	331
1853	D1713	*C. jejuni* subsp. *jejuni*	Human	USA		1454, 1016	331
1854	D1916	*C. jejuni* subsp. *jejuni*	Human	USA		1454, 1016	331
1855	D2583	*C. jejuni* subsp. *jejuni*	Human	USA		1454, 1016	331
1856	D2589	*C. jejuni* subsp. *jejuni*	Human	Scotland		1454, 1016	331
1859	L17	*C. jejuni* subsp. *jejuni*	Unknown	Unknown	Lior 17; HS:4,16	1454, 1016	331
1860	L18	*C. jejuni* subsp. *jejuni*	Unknown	Unknown	Lior 18, HS:55	1454, 1016	331
1861	L19	*C. jejuni* subsp. *jejuni*	Unknown	Unknown	Lior 19, HS:42,15	1454, 1016	331
1862	NCTC 11168	*C. jejuni* subsp. *jejuni*	Human	UK	HS:2; Sequence strain	1454, 1016	331
1863	81116	*C. jejuni* subsp. *jejuni*	Human	UK		1454, 1016	331
1864	81-176	*C. jejuni* subsp. *jejuni*	Human	USA (MN)	HS:23,36; Sequence strain	1454, 1016	331
1866	D1917	*C. jejuni* subsp. *jejuni*	Chicken	USA		1454, 1016	331
1881	ATCC 33291	*C. jejuni* subsp. *jejuni*	Human	USA (CO)		1454, 1016	331
1892	K21	*C. jejuni* subsp. *jejuni*	Chicken	USA		1454, 1016	331
1893	K22	*C. jejuni* subsp. *jejuni*	Chicken	USA		1454, 1016	331
1894	K23	*C. jejuni* subsp. *jejuni*	Chicken	USA		1454, 1016	331
1895	K24	*C. jejuni* subsp. *jejuni*	Chicken	USA		1454, 1016	331
1910	K20	*C. jejuni* subsp. *jejuni*	Chicken	USA		1454, 1016	331
2226	72237	*C. jejuni* subsp. *jejuni*	Chicken	USA		1454, 1016	331
2227	72522	*C. jejuni* subsp. *jejuni*	Chicken	USA		1454, 1016	331
2229	72737	*C. jejuni* subsp. *jejuni*	Chicken	USA		1454, 1016	331
2232	72927	*C. jejuni* subsp. *jejuni*	Chicken	USA		1454, 1016	331
2233	72934	*C. jejuni* subsp. *jejuni*	Chicken	USA		1454, 1016	331
2239	75009	*C. jejuni* subsp. *jejuni*	Chicken	USA		1454, 1016	331
2240	75059	*C. jejuni* subsp. *jejuni*	Chicken	USA		1454, 1016	331

2241	75284	*C. jejuni* subsp. *jejuni*	Chicken	USA		1454, 1016	331
2769		*C. jejuni* subsp. *jejuni*	Chicken	USA		1454, 1016	331
2872		*C. jejuni* subsp. *jejuni*	Chicken	USA		1454, 1016	331
2873		*C. jejuni* subsp. *jejuni*	Chicken	USA		1454, 1016	331
3145	HB93-13	*C. jejuni* subsp. *jejuni*	Human	China	HS:19; GBS isolate	1454, 1016	331
3146	HB93-29	*C. jejuni* subsp. *jejuni*	Human	China	HS:19; GBS isolate	1454, 1016	331
3147	INP7	*C. jejuni* subsp. *jejuni*	Human	Mexico	HS:19; GBS isolate	1454, 1016	331
3148	INP21	*C. jejuni* subsp. *jejuni*	Human	Mexico	HS:19; GBS isolate	1454, 1016	331
3149	INP59	*C. jejuni* subsp. *jejuni*	Human	Mexico	HS:41; GBS isolate	1454, 1016	331
3193	290.94	*C. jejuni* subsp. *jejuni*	Human	S. Africa	HS:41; GBS isolate	1454, 1016	331
3194	285.94	*C. jejuni* subsp. *jejuni*	Human	S. Africa		1454, 1016	331
3196	233.94	*C. jejuni* subsp. *jejuni*	Human	S. Africa	HS:41; GBS isolate	1454, 1016	331
3197	308.95	*C. jejuni* subsp. *jejuni*	Human	S. Africa	HS:41; GBS isolate	1454, 1016	331
3198	367.95	*C. jejuni* subsp. *jejuni*	Human	S. Africa	HS:41; GBS isolate	1454, 1016	331
3199	370.95	*C. jejuni* subsp. *jejuni*	Human	S. Africa	HS:41; GBS isolate	1454, 1016	331
3200	302.96	*C. jejuni* subsp. *jejuni*	Human	S. Africa	HS:33	1454, 1016	331
3201	378.96	*C. jejuni* subsp. *jejuni*	Human	S. Africa	HS:41	1454, 1016	331
3202	386.96	*C. jejuni* subsp. *jejuni*	Human	S. Africa	HS:41	1454, 1016	331
3203	16.97	*C. jejuni* subsp. *jejuni*	Human	S. Africa	HS:12	1454, 1016	331
3204	20.97	*C. jejuni* subsp. *jejuni*	Human	S. Africa	HS:12	1454, 1016	331
3205	199.97	*C. jejuni* subsp. *jejuni*	Human	S. Africa	HS:41	1454, 1016	331
3206	231.97	*C. jejuni* subsp. *jejuni*	Human	S. Africa	HS:8,17	1454, 1016	331
3207	250.97	*C. jejuni* subsp. *jejuni*	Human	S. Africa	HS:41	1454, 1016	331
3208	1.98	*C. jejuni* subsp. *jejuni*	Human	S. Africa	HS:21	1454, 1016	331
3209	24.98	*C. jejuni* subsp. *jejuni*	Human	S. Africa	HS:12	1454, 1016	331
3210	242.98	*C. jejuni* subsp. *jejuni*	Human	S. Africa	HS:41	1454, 1016	331
3211	96	*C. jejuni* subsp. *jejuni*	Human	S. Africa		1454, 1016	331
3264	17387	*C. jejuni* subsp. *jejuni*	Human	Canada	GBS isolate	1454, 1016	331
3265	98-1718	*C. jejuni* subsp. *jejuni*	Human	Canada	GBS isolate	1454, 1016	331
3266	17714	*C. jejuni* subsp. *jejuni*	Human	Canada	GBS isolate	1454, 1016	331
3405		*C. jejuni* subsp. *jejuni*	Unknown	Unknown	HS:1 reference strain	1454, 1016	331
3406		*C. jejuni* subsp. *jejuni*	Unknown	Unknown	HS:2 reference strain	1454, 1016	331
3407		*C. jejuni* subsp. *jejuni*	Unknown	Unknown	HS:3 reference strain	1454, 1016	331
3408		*C. jejuni* subsp. *jejuni*	Unknown	Unknown	HS:4 reference strain	1454, 1016	331
3409		*C. jejuni* subsp. *jejuni*	Unknown	Unknown	HS:5 reference strain	1454, 1016	331

3410	*C. jejuni* subsp. *jejuni*	Unknown	Unknown	HS:6 reference strain	1454, 1016	331
3411	*C. jejuni* subsp. *jejuni*	Unknown	Unknown	HS:7 reference strain	1454, 1016	331
3412	*C. jejuni* subsp. *jejuni*	Unknown	Unknown	HS:8 reference strain	1454, 1016	331
3413	*C. jejuni* subsp. *jejuni*	Unknown	Unknown	HS:9 reference strain	1454, 1016	331
3414	*C. jejuni* subsp. *jejuni*	Unknown	Unknown	HS:10 reference strain	1454, 1016	331
3415	*C. jejuni* subsp. *jejuni*	Unknown	Unknown	HS:11 reference strain	1454, 1016	331
3416	*C. jejuni* subsp. *jejuni*	Unknown	Unknown	HS:13 reference strain	1454, 1016	331
3417	*C. jejuni* subsp. *jejuni*	Unknown	Unknown	HS:16 reference strain	1454, 1016	331
3418	*C. jejuni* subsp. *jejuni*	Unknown	Unknown	HS:17 reference strain	1454, 1016	331
3419	*C. jejuni* subsp. *jejuni*	Unknown	Unknown	HS:18 reference strain	1454, 1016	331
3421	*C. jejuni* subsp. *jejuni*	Unknown	Unknown	HS:22 reference strain	1454, 1016	331
3422	*C. jejuni* subsp. *jejuni*	Unknown	Unknown	HS:23 reference strain	1454, 1016	331
3423	*C. jejuni* subsp. *jejuni*	Unknown	Unknown	HS:27 reference strain	1454, 1016	331
3424	*C. jejuni* subsp. *jejuni*	Unknown	Unknown	HS:29 reference strain	1454, 1016	331
3425	*C. jejuni* subsp. *jejuni*	Unknown	Unknown	HS:32 reference strain	1454, 1016	331
3426	*C. jejuni* subsp. *jejuni*	Unknown	Unknown	HS:35 reference strain	1454, 1016	331
3427	*C. jejuni* subsp. *jejuni*	Unknown	Unknown	HS:36 reference strain	1454, 1016	331
3428	*C. jejuni* subsp. *jejuni*	Unknown	Unknown	HS:37 reference strain	1454, 1016	331
3429	*C. jejuni* subsp. *jejuni*	Unknown	Unknown	HS:38 reference strain	1454, 1016	331
3430	*C. jejuni* subsp. *jejuni*	Unknown	Unknown	HS:41 reference strain	1454, 1016	331
3431	*C. jejuni* subsp. *jejuni*	Unknown	Unknown	HS:44 reference strain	1454, 1016	331
3432	*C. jejuni* subsp. *jejuni*	Unknown	Unknown	HS:45 reference strain	1454, 1016	331
3433	*C. jejuni* subsp. *jejuni*	Unknown	Unknown	HS:50 reference strain	1454, 1016	331
3434	*C. jejuni* subsp. *jejuni*	Unknown	Unknown	HS:52 reference strain	1454, 1016	331
3435	*C. jejuni* subsp. *jejuni*	Unknown	Unknown	HS:53 reference strain	1454, 1016	331
3436	*C. jejuni* subsp. *jejuni*	Unknown	Unknown	HS:57 reference strain	1454, 1016	331
3437	*C. jejuni* subsp. *jejuni*	Unknown	Unknown	HS:58 reference strain	1454, 1016	331
3438	*C. jejuni* subsp. *jejuni*	Unknown	Unknown	HS:60 reference strain	1454, 1016	331
3439	*C. jejuni* subsp. *jejuni*	Unknown	Unknown	HS:62 reference strain	1454, 1016	331
3440	*C. jejuni* subsp. *jejuni*	Unknown	Unknown	HS:63 reference strain	494	331
3441	*C. jejuni* subsp. *jejuni*	Unknown	Unknown	HS:64 reference strain	1454, 1016	331
3442	*C. jejuni* subsp. *jejuni*	Unknown	Unknown	HS:65 reference strain	1454, 1016	331
3664	*C. jejuni* subsp. *jejuni*	Goose	USA (CA)		1454, 1016	331
3665	*C. jejuni* subsp. *jejuni*	Goose	USA (CA)		1454, 1016	331
3666	*C. jejuni* subsp. *jejuni*	Goose	USA (CA)		1454, 1016	331

3667		*C. jejuni* subsp. *jejuni*	Goose	USA (CA)		1454, 1016	331
3668		*C. jejuni* subsp. *jejuni*	Goose	USA (CA)		1454, 1016	331
3669		*C. jejuni* subsp. *jejuni*	Goose	USA (CA)		1454, 1016	331
3670		*C. jejuni* subsp. *jejuni*	Goose	USA (CA)		1454, 1016	331
3672		*C. jejuni* subsp. *jejuni*	Goose	USA (CA)		1454, 1016	331
3673		*C. jejuni* subsp. *jejuni*	Goose	USA (CA)		1454, 1016	331
3674		*C. jejuni* subsp. *jejuni*	Goose	USA (CA)		1454, 1016	331
4767	6893	*C. jejuni* subsp. *jejuni*	Human	Canada (BC)		1454, 1016	331
4768	6894	*C. jejuni* subsp. *jejuni*	Human	Canada (BC)		1454, 1016	331
4769	6895	*C. jejuni* subsp. *jejuni*	Human	Canada (BC)		1454, 1016	331
4770	6574	*C. jejuni* subsp. *jejuni*	Human	Canada (ON)		1454, 1016	331
4771	6575	*C. jejuni* subsp. *jejuni*	Human	Canada (ON)		1454, 1016	331
4772	6576	*C. jejuni* subsp. *jejuni*	Human	Canada (ON)		1454, 1016	331
3663	NCTC 13004	*C. lanienae*	Unknown	UK	Type strain	None	None
1887	ATCC 35223	*C. lari*	Human	Unknown		1454	235
1890	ATCC 43675	*C. lari*	Human	Unknown		1454	235
2100	D67	*C. lari*	Human	USA		1454	235
2808	ATCC 35221	*C. lari*	Unknown	Unknown		1454	235
2809	ATCC 35222	*C. lari*	Unknown	Unknown		1454	235
2810	ATCC 43675	*C. lari*	Unknown	Unknown		1454	235
2816	LMG 8844	*C. lari*	Seawater	UK		1454	235
2817	LMG 9152	*C. lari*	Horse	Sweden		1454	235
2818	LMG 9253	*C. lari*	Human	Unknown		1454	235
2819	LMG 9887	*C. lari*	Seagull	Unknown		1454	235
2820	LMG 9888	*C. lari*	Seagull	Unknown		1454	235
2821	LMG 9889	*C. lari*	Seagull	Unknown		1454	235
2822	LMG 9913	*C. lari*	Human	Unknown		1454	235
2823	LMG 9914	*C. lari*	Human	Unknown		1454	235
2824	LMG 11251	*C. lari*	Unknown	Unknown		1454	235
2825	LMG 11760	*C. lari*	Human	Canada		1454	235
2826	LMG 14338	*C. lari*	Human	Belgium		1454	235
3659	NCTC 11845	*C. lari*	River water	UK	UPTC	None	235
3660	NCTC 11928	*C. lari*	River water	UK	UPTC	1454	235
3661	NCTC 11937	*C. lari*	Unknown	UK	UPTC	None	235
4110	CCUG 22395	*C. lari*	Human	France	UPTC	1454	235

3233	ATCC 43265	*C. mucosalis*	Porcine	Unknown		None	None
3234	ATCC 43264	*C. mucosalis*	Porcine	Unknown	Type strain	~1100	None
3235		*C. mucosalis*	Unknown	Unknown		None	None
4113	CCUG 21559	*C. mucosalis*	Porcine	Scotland	Serotype A	None	None
4114	CCUG 23201	*C. mucosalis*	Porcine	Scotland	Serotype B	None	None
3267	ATCC 33238	*C. rectus*	Human	Unknown	Type strain	None	None
3277	ATCC 51146	*C. showae*	Human	Japan	Type strain	None	None
4121	CCUG 20703	*C. sputorum* bv. *faecalis*	Sheep	UK		1454	None
4120	LMG 11764	*C. sputorum* bv. *paraureolyticus*	Human	Canada		None	None
3237	ATCC 51146	*C. sputorum* bv. *sputorum*	Human	Brazil		1454	None
4119	86.92	*C. sputorum* bv. *sputorum*	Human	S. Africa		1454	None
1488	ATCC 49815	*C. upsaliensis*	Human	Canada		1454, 1016	206
2089	D1137	*C. upsaliensis*	Sheep	Unknown		1454, 1016	206
2092	D1673	*C. upsaliensis*	Human	Unknown		1454, 1016	206
2093	D1178	*C. upsaliensis*	Human	USA		1454, 1016	206
2094	D2237	*C. upsaliensis*	Human	USA		1454, 1016	206
3195	300.94	*C. upsaliensis*	Human	S. Africa		1016	206
3776	365.96	*C. upsaliensis*	Human	S. Africa		1016	206
3777	3.97	*C. upsaliensis*	Human	S. Africa		None	206
3778	5.97	*C. upsaliensis*	Human	S. Africa		1454, 1016	206
3779	1.02	*C. upsaliensis*	Human	S. Africa		1016	206
3780	18.02	*C. upsaliensis*	Human	S. Africa		1016	206
3781	21.02	*C. upsaliensis*	Human	S. Africa		1016	206
3782	34.02	*C. upsaliensis*	Human	S. Africa		None	206
3783	68.02	*C. upsaliensis*	Human	S. Africa		1016	206
3784	162.02	*C. upsaliensis*	Human	S. Africa		1016	206
3785	26.03	*C. upsaliensis*	Human	S. Africa		1016	206
3786	37.03	*C. upsaliensis*	Human	S. Africa		1016	206
3810		*C. upsaliensis*	Feline	USA (CA)		1454, 1016	206
3937	003	*C. upsaliensis*	Human	USA (CA)		1454, 1016	206
3939	021	*C. upsaliensis*	Human	USA (CA)		1454, 1016	206
3940	054	*C. upsaliensis*	Human	USA (CA)		1454, 1016	206
3941	058	*C. upsaliensis*	Human	USA (CA)		1016	206
3942	0675	*C. upsaliensis*	Human	USA (CA)		1016	206

3943	1419	*C. upsaliensis*	Human	USA (CA)		1016	206
3944	3846	*C. upsaliensis*	Human	USA (CA)		1454, 1016	206
3945	1882	*C. upsaliensis*	Canine	USA (CA)		1454, 1016	206
3946	1883	*C. upsaliensis*	Canine	USA (CA)		1016	206
3947	1884	*C. upsaliensis*	Canine	USA (CA)		1454, 1016	206
3948	1885	*C. upsaliensis*	Canine	USA (CA)		1016	206
3949	2041	*C. upsaliensis*	Canine	USA (CA)		1454, 1016	206
3950	2042A	*C. upsaliensis*	Canine	USA (CA)		1454, 1016	206
4039	181.00	*C. upsaliensis*	Unknown	S. Africa		1016	206
4040	101.01	*C. upsaliensis*	Unknown	S. Africa		1016	206
4042	106.01	*C. upsaliensis*	Unknown	S. Africa		1016	206
4043	109.01	*C. upsaliensis*	Unknown	S. Africa		1016	206
4044	112.01	*C. upsaliensis*	Unknown	S. Africa		1016	206
4046	130.01	*C. upsaliensis*	Unknown	S. Africa		1016	206
4047	155.01	*C. upsaliensis*	Unknown	S. Africa		1016	206
4048	3.97	*C. upsaliensis*	Unknown	S. Africa		1016	206
4049	283.02	*C. upsaliensis*	Unknown	S. Africa		1016	206
4051	78.03	*C. upsaliensis*	Unknown	S. Africa		1454, 1016	206
4055	173.92	*C. upsaliensis*	Human	S. Africa		1454, 1016	206
4058	420.92	*C. upsaliensis*	Unknown	S. Africa		1016	206
4059	430.92	*C. upsaliensis*	Human	S. Africa		1016	206
4061	131.01	*C. upsaliensis*	Human	S. Africa		1016	206
4062	152.01	*C. upsaliensis*	Human	S. Africa		1016	206
4063	164.01	*C. upsaliensis*	Human	S. Africa		1016	206
4064	169.01	*C. upsaliensis*	Human	S. Africa		1016	206
4065	171.01	*C. upsaliensis*	Human	S. Africa		1016	206
4066	118.03	*C. upsaliensis*	Human	S. Africa		1016	206
4068	137.03	*C. upsaliensis*	Human	S. Africa		1016	206
4069	145.03	*C. upsaliensis*	Human	S. Africa		None	206
4123	CCUG 19559	*C. upsaliensis*	Human	UK		1454, 1016	206
4124	CCUG 19607	*C. upsaliensis*	Canine	Sweden		1454, 1016	206
4133	CCUG 14913	*C. upsaliensis*	Canine	Sweden	Type strain	1454, 1016	206
4134	CCUG 23017	*C. upsaliensis*	Human	France		1016	206
4135	CCUG 20818	*C. upsaliensis*	Human	USA		1454, 1016	206
4136	CCUG 33890	*C. upsaliensis*	Canine/Feline	Sweden		1016	206

4137	Abdn F511	*C. upsaliensis*	Unknown	Scotland	1454, 1016	206
4244	LMG 9104	*C. upsaliensis*	Human	Belgium	1016	206
4245	LMG 9108	*C. upsaliensis*	Human	Belgium	1016	206
4246	LMG 9114	*C. upsaliensis*	Human	Belgium	1016	206
4248	LMG 9125	*C. upsaliensis*	Human	Belgium	1016	206
4249	LMG 9129	*C. upsaliensis*	Human	Belgium	1016	206
4250	LMG 9140	*C. upsaliensis*	Human	Belgium	1016	206
4251	LMG 9222	*C. upsaliensis*	Human	Belgium	1016	206
4252	LMG 9226	*C. upsaliensis*	Human	Belgium	1016	206
4253	LMG 9230	*C. upsaliensis*	Human	Belgium	1016	206
4255	LMG 9240	*C. upsaliensis*	Human	Belgium	1454, 1016	206
4256	LMG 9261	*C. upsaliensis*	Human	Belgium	1016	206
4257	LMG 9265	*C. upsaliensis*	Human	Belgium	1016	206
4258	LMG 9269	*C. upsaliensis*	Human	Belgium	1016	206

a. Band sizes are in bp.
